# Design, characterisation and validation of a haptic interface based on twisted string actuation

**DOI:** 10.3389/frobt.2022.977367

**Published:** 2022-09-16

**Authors:** Valeria Skvortsova, Simeon Nedelchev, Joshua Brown, Ildar Farkhatdinov, Igor Gaponov

**Affiliations:** ^1^ Center for Technologies in Robotics and Mechatronics Components, Innopolis University, Innopolis, Russia; ^2^ School of Electronic Engineering and Computer Science, Queen Mary University of London, London, United Kingdom

**Keywords:** haptic interface, twisted string actuator, human-robot interface, cable-driven system, robot control (RC)

## Abstract

This paper presents the design and experimental characterisation of a wrist haptic interface based on a twisted string actuator. The interface is designed for controlled actuation of wrist flexion/extension and is capable of rendering torque feedback through a rotary handle driven by the twisted string actuator and spring-loaded cable mechanisms. The interface was characterised to obtain its static and dynamic haptic feedback rendering capabilities. Compliance in the spring and actuation mechanism makes the interface suitable for smooth rendering of haptic feedback of large magnitudes due to the high motion transmission ratio of the twisted strings. Haptic virtual wall rendering capabilities are demonstrated.

## 1 Introduction

Robots are efficient tools to study human neuromechanics Klein et al. (2013); Melendez-Calderon et al. (2014); Huang et al. (2020) and provide haptic feedback in physical human-machine interaction applications Farkhatdinov et al. (2019); Gonzalez et al. (2015); Trinitatova et al. (2019). Robotic systems designed for these applications typically use electromagnetic actuation to produce forces and apply them to the user’s body, while some haptic interfaces also use pneumatic, hydraulic, piezoelectric, and other actuation technologies. Combining actuators and transmissions of various types helps robotic devices generate desired dynamic characteristics for physical human-robot interaction, and therefore when selecting the actuator one must take into account the levels of forces and torques, achievable stiffness, response time, control precision, and other performance indicators afforded by each actuation technology.

Twisted string actuation (TSA) has emerged in the past decade as one of the more efficient methods to transmit motion in robotic mechanisms Zhang et al. (2019). The operation of actuators built on this principle is based on the phenomenon in which the physical twisting of strings or cables around their longitudinal axis causes them to contract. This effect can be then used to convert rotational motion into linear one with high transmission ratios. TSAs have a unique set of advantages which makes them an attractive choice for haptic and assistive robotic devices. These benefits include inherent compliance, low weight, flexibility in cable routing, and comparatively high efficiency. Several implementations for TSA-based robotic exoskeletons have been described recently for elbow (Meattini et al., 2017) and hip (Seong et al., 2020) joints in load carrying/lifting tasks, for haptic rendering for finger-worn systems (Hosseini et al., 2018; Chossat et al., 2019) and virtual wall rendering with grounded haptic interfaces (Van and Harders, 2017; Feenstra et al., 2021). User studies by Hosseini et al. (2018) and Chossat et al. (2019) have demonstrated the capabilities of TSA to render haptic effects (haptic wall, stiffness) but the proposed interfaces were used to provide force feedback only to finger joints that require less powerful actuation. The development of more powerful haptic interfaces with TSA is a challenging design and control engineering problem as higher haptic rendering performance (stiffness, dynamic response) requires faster and stronger TSA mechanisms.

This paper presents a preliminary design and characterisation of a wrist haptic device based on TSA. Compared to existing TSA-based haptic interfaces (Van and Harders, 2017; Hosseini et al., 2018; Chossat et al., 2019; Feenstra et al., 2021) the proposed device has been specifically developed for a human wrist support that requires significant torque generation capability. Robotic systems to assist human wrist flexion and extension require high actuation torques and control bandwidth. The use of single-joint haptic interfaces provides important advantages to studying human motor control and developing new rehabilitation and assistive robots, as it enables us to analyse human motor commands precisely by focusing on one group of antagonist muscles. For example, single joint (flexion/extension) wrist haptic interfaces have been successfully used for human neuromechanics research as they allow efficient acquisition and analysis of arm kinematics, muscle activation and brain (motor cortex) activation (McClelland et al., 2021; Farkhatdinov et al., 2015; Wilhelm et al., 2016; Melendez-Calderon et al., 2011; Farkhatdinov et al., 2022; Perez et al., 2022). Single degree-of-freedom robots can be efficiently used for neuromotor rehabilitation, as well (Milot et al., 2013; Saita et al., 2017; Kubota et al., 2019), and hence the development of novel single degree-of-freedom robots and haptic interfaces like the one presented in this paper has a direct impact on the advancement of neurorehabilitation technology.

**FIGURE 1:**
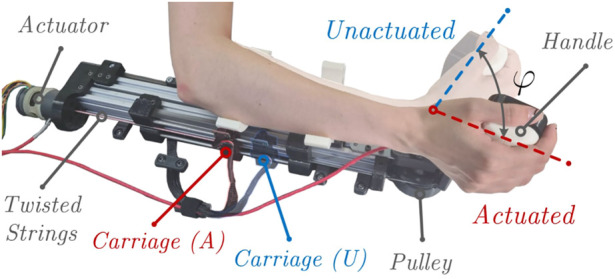
Proposed TSA-based wrist haptic interface in the unactuated (the strings are untwisted) and actuated (the strings are twisted) states.

The designed interface is shown in Figure 1. A user interacts with the robot through a wrist handle that is actuated by TSA while the forces produced by twisted strings are converted into flexion/extension torques that are subsequently applied to the human’s wrist. In this paper, we describe device design, report on the static and dynamic characterisation of the developed human-robot interface, and present preliminary experimental results of the virtual wall rendering. Practical validation of the developed TSA-based device demonstrated that it could achieve a sufficient range of dynamic torques for wrist haptic feedback, with motion frequencies reaching up to 10 Hz. To the best of our knowledge, this is the first evaluation of this kind in a TSA-based wrist haptic interface to study the system’s force transmission capabilities at high frequencies. Experimental results matched the theoretical model with sufficient accuracy, and the analysis presented in this paper will lay the groundwork for the design of the next generation of haptic interfaces based on TSA.

The next section of the paper reviews the kinematics of twisted strings and describes the kinematics and dynamics of the developed TSA-based haptic interface. Section 3 describes the static and dynamic characterisation of the device, and the paper is concluded with the experimental evaluation of the device and relevant discussion.

## 2 TSA-based haptic interface

### 2.1 Overview of twisted string kinematics

**FIGURE 2:**
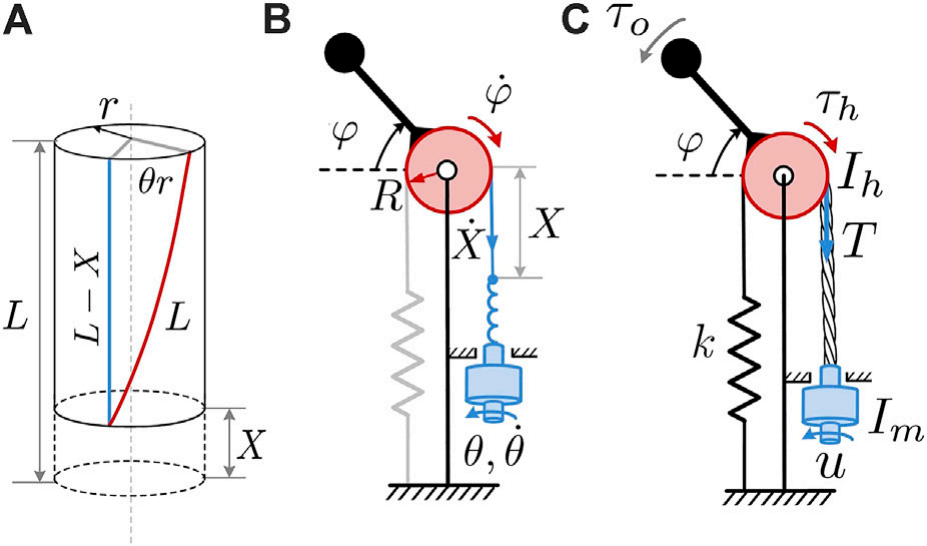
**(A)** A section of a twisted string represented by a cylinder, **(B)** schematic diagram of device kinematics, **(C)** schematic diagram for dynamics calculations.

In TSA, one end of a string is attached coaxially to the motor shaft while the other end is fixed at the robot’s end-effector or payload. Twisting the strings with the motor results in their contraction, thereby driving the end-effector. According to the conventional model of twisted strings, Palli et al. (2012); Gaponov et al. (2013), when a cable of length *L* and radius *r* is twisted at an angle *θ*, it forms a helix and contracts by length *X*, as shown in Figure 2A. For the resulting right triangle with the sides (*L* − *X*), *θr*, and *L* one can drive the geometric constraint of twisted string as:

$$\theta^{2}r^{2}+{\left(L-X\right)}^{2}-L^{2}=0.$$ (1)

Eq. 1can be used to calculate the displacement *X* produced by a string twisted by some angle *θ*. To obtain the corresponding velocity relationships, one can simply differentiate Eq. 1 with respect to time to obtain a general expression of the form

$$\dot{X}=J\left(\theta ,X\right)\dot{\theta }$$ (2)

where J denotes the Jacobian of twisted strings, $\dot{\theta }$ and $\dot{X}$ stands for angular and linear velocities. Hereinafter, we will omit the arguments (*θ*, *X*) in Jacobian for the sake of brevity. It is worth noting that the twisted strings Jacobian may be represented in various ways, depending on the availability of the motor or end-effector measurements (*θ* and *X*, respectively). For instance, one can use the following expressions interchangeably:

$$J=\frac{\theta r^{2}}{L-X}=\frac{\theta r^{2}}{\sqrt{L^{2}-\theta^{2}r^{2}}}=\frac{r\sqrt{L^{2}-{\left(L-X\right)}^{2}}}{L-X}$$ (3)

### 2.2 Kinematics of the proposed haptic interface

A schematic diagram of the kinematics of the proposed TSA-based haptic interface is shown in Figure 2B. An electric motor’s shaft is connected to a set of strings (shown in blue). The other ends of the strings are connected to a pulley (shown in red) by means of an untwisted cable, while a handle is rigidly attached to the pulley.

The motor twists the strings and causes their contraction, *X*, which subsequently causes angular displacement of the pulley *φ* (wrist rotation). The pulley is of constant radius *R* and has a linear extension spring attached to its opposite side to facilitate the restoring motion when the cable is untwisted. The stiffness of the spring and position of the cable attachments are selected such that a neutral position of the pulley and wrist is aligned with the cables/strings orientation and set to *φ* = 0. Noting that *φ* = *X*/*R*, one may rewrite Eq. 1 as:

$$\theta^{2}r^{2}+{\left(L-\varphi R\right)}^{2}-L^{2}=0.$$ (4)

Then we can explicitly express pulley’s (wrist handle) angle, *φ*, as the function of motor’s angle, *θ*, as

$$\varphi \left(\theta \right)=\frac{1}{R}\left(L-\sqrt{L^{2}-\theta^{2}r^{2}}\right),$$ (5)

and its angular velocity

$$\dot{\varphi }\left(\theta \right)=\frac{1}{R}J\dot{\theta }=J_{d}\dot{\theta }$$ (6)

with $J_{d}\equiv \frac{1}{R}J$ denoting device’s Jacobian as

$$J_{d}=\frac{\theta r^{2}}{R\left(L-\varphi R\right)}=\frac{\theta r^{2}}{R\sqrt{L^{2}-\theta^{2}r^{2}}}=\frac{r\sqrt{L^{2}-{\left(L-\varphi R\right)}^{2}}}{R\left(L-\varphi R\right)}$$ (7)

### 2.3 Dynamic model of the interface

In the proposed device, the motor generates torque *u* that twists the strings and rotates the pulley in the clockwise direction in Figure 2C. Extension springs are connected to the opposite end of the pulley to facilitate the returning motion and to ensure that the cables are always under tension. As a result, the string is subjected to tension *T* that is converted to the torque on the handle *τ*_*h*_ = *T* ⋅ *R*. This torque is countered by the corresponding spring torque *kRφ* from the linear extension spring. During operation a user exerts torque *τ*_*o*_ by acting on the wrist handle that is rigidly connected to the pulley.

Disregarding a user’s hand dynamics and assuming that the mass of the strings, cables, and springs is negligible, the interface can be modeled with two dynamic subsystems: the motor and the pulley with handle. Their equations of motion are

$$\left\{\begin{array}{c} u=I_{m}\ddot{\theta }+J\left(\theta \right)T+b_{\theta }\dot{\theta }+\tau_{d},\;\\ \tau_{h}=T\cdot R=I_{h}\ddot{\varphi }+\tau_{o}+kR\varphi \; \end{array}\right.$$ (8)

where *b*_*θ*_ represents the viscous friction coefficient on the motor side, the terms *I*_*m*_ and *I*_*h*_ stand for the moments of inertia of the motor shaft and the handle-pulley system respectively, while the term *τ*_*d*_ represents the collective effects of all other forces at play inside the TSA system such as dry friction, string jamming and external disturbance applied to the payload. It should be specifically noted that the term *τ*_*d*_ also describes the effects of variable string stiffness that decreases with twisting, as we have investigated in our previous studies (Popov et al., 2014). The effects of increasing compliance of twisted strings may become significant at high values of relative string contraction, however, this is not the case for the developed system that features comparatively long strings and small contraction magnitudes (by design). Lastly, the developed feedback controller compensates for any model inaccuracies thanks to direct measurements of the handle’s angle and torque, yielding positioning accuracy which was deemed satisfactory for our application. For more information and details on the derivation of the components of the *τ*_*d*_ torque and particularly the ones responsible for energy losses within the strings due to their intrinsic compliance, please refer to our previous work (Nedelchev et al., 2020). Equations of motion Eq. 8 can be used to obtain transfer functions defining the relationship between the motor’s input position and torque and the output handle’s position and torque, however these are valid only for a limited range of motion of the interface as the term J depends on *θ* or *X* as suggested by Eq. 3.

### 2.4 Design of the haptic interface

**FIGURE 3:**
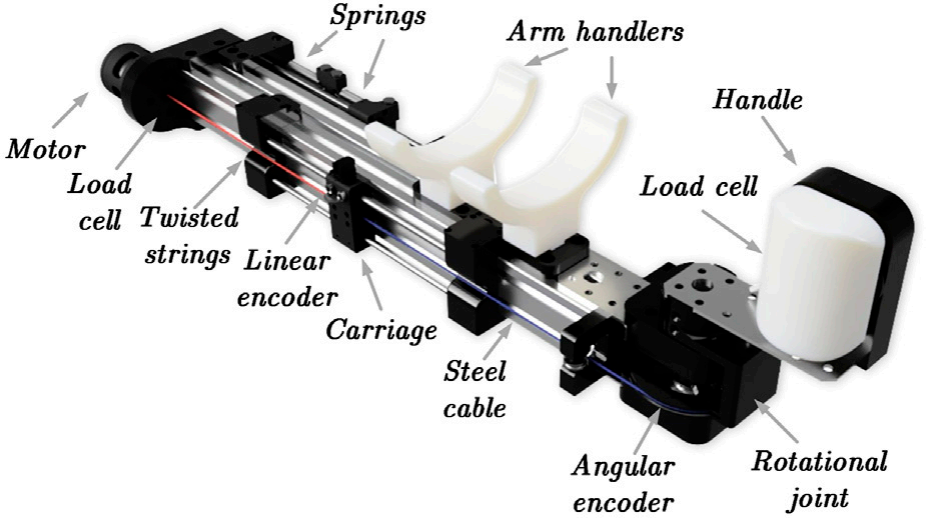
A CAD model of the proposed TSA-based wrist haptic interface.

An experimental haptic device prototype realizing the kinematical scheme shown in Figure 2B was designed and manufactured. A CAD model of the proposed TSA-based haptic wrist interface with its main components is shown in Figure 3, while the actual prototype is presented in Figure 1. The device consists of a rigid metal frame which serves as a base for a TSA actuation module, a sliding cable mechanism, springs attachment, human arm supports, and bearings for the wrist handle joint.

The wrist handle joint is driven by a TSA module that houses a DC motor to twist the strings. The other end of the string is attached to a carriage that moves along the guides supported by linear sleeve bearings, while its position is measured by a dedicated linear encoder. The carriage’s motion is transferred to the rotational joint through a steel cable. We use a pair of strings with untwisted length of 320 mm and diameter of 0.8 mm. The strings’ length is selected in such that their relative contraction by 30% of the initial length corresponds to 180° rotation of the handle. The springs are attached to the side of the pulley opposite to the twisted strings to facilitate the restoring motion of the TSA, thus making the joint bidirectional. The range of motion of the wrist handle was approximately 150°, which fully covers the natural range of the human’s wrist flexion and extension (140°).

An outrunner BLDC motor driven by the integrated driver (Gyems DRC06) and equipped with a 14-bit absolute encoder was used to drive the TSA. An optical incremental encoder (Avago HEDS9040 2048 CPT) was integrated in the robot’s wrist joint to measure the handle’s angular position (*φ*). A linear encoder with the resolution of 18 *μ*m (Avago H9740-1 360 LPI) was used to measure the actual string contraction (*X*). A load cell (Futek LTH300 50 lb) was installed in the handle to provide the measurements of the interaction forces between the handle and the user’s palm (*τ*_*h*_ − *τ*_◦_). Another load cell was installed between the motor and a thrust bearing that the former is pushing against to measure the strings’ tension (*T*). The sampling rate for motor commands and sensory data acquisition was 500 Hz.

## 3 Static characterisation

### 3.1 Methods

The aim of this experiment was to investigate the kinematics of the proposed haptic interface described by model Eq. 4. In particular, the first objective was to characterise how angular displacement of the wrist joint *φ* changes with cable twisting *θ*. The second objective was to characterise the static TSA force to handle torque transmission gain.

We conducted several experiments with the prototype during which the motor was controlled to follow a periodic reference position signal of the form

$$\theta =\frac{A}{2}\left(1-\cos wt\right)$$ (9)

with the motor shaft rotation magnitude *A* = 300 rad (approx. 48 full turns) chosen such that the handle rotated by just over 150° (requiring roughly 25% of relative string contraction), while the oscillation frequency *w* was set at 0.15 rad/s (simulating quasi-static conditions). During the experiment, we measured the carriage’s and handle’s positions, motor current, and generated string tension. With these data, we were able to plot the kinematic relationships and calculate the transmission ratio of the device *K* for the whole range of the handle’s motion using the simple formula

$$K=\left\|\frac{T}{u}\right\|.$$ (10)

### 3.2 Results

**FIGURE 4:**
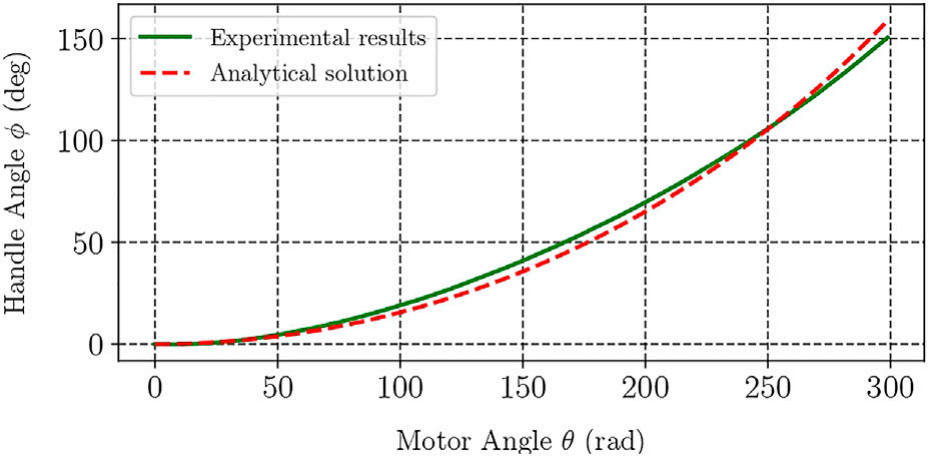
Theoretical and experimental handle angles versus the angle of twisting in the proposed haptic interface.

The results of the kinematic evaluation of the device are presented in Figure 4 which depicts the experimental and theoretical curves of handle’s angle as a function of motor angle. The analytical solution was calculated with the help of the device’s kinematics model Eq. 5. Analysis of the experimental data showed that the mean squared error between theoretical and practical values was under 1.3%, which was considered satisfactory in this study.

**FIGURE 5:**
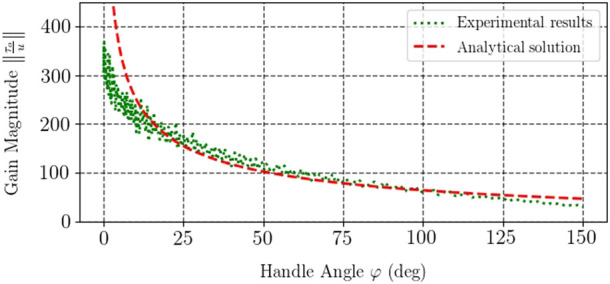
Force transmission ratio of the device for various handle angles.

Figure 5 depicts theoretical and experimental curves of the transmission ratio of the developed interface. The analytical relationship was calculated with the model Eq. 7 and corresponds to the Jacobian inverse. Experimental data were obtained using the model Eq. 10 and accounting for the effect of dry friction inside the motor whose magnitude was measured to be about 9 mNm. Dry friction was found using the data of idle motor current when no load was applied. Analysis of the experimental data showed that the theoretical and practical transmission ratios differed by 2.9% in terms of RMSE, which again was deemed satisfactory for the developed prototype.

## 4 Dynamic characterisation

### 4.1 Methods

Dynamic characterisation of haptic devices most often implies investigating their transfer function (e.g., the ratio between the output and input forces or torques) for a range of frequencies. This investigation can generally be done for any operating point of the device if the system can be modeled as a linear one. Twisted strings, however, exhibit nonlinear kinematics, as becomes evident from Figure 4 and Figure 5, and therefore one cannot perform dynamic characterisation of a TSA-based device at an arbitrary operation point and extend these findings to the whole operation range of the system. Thus, in the dynamical characterisation experiment we aimed to find the response of the device at different angles of the handle.

The experiment was conducted without springs to avoid any interference due to their nonlinearity and non-negligible mass. A rigid wall was placed perpendicular to the load cell at the handle. During the experiment, we slowly brought the handle to the wall and pressed into it by rotating the motor with the strings further. Once a certain force threshold (15 N in our case) was achieved, the motor was controlled in the torque mode using a reference torque chirp signal:

$$u=u_{0}+A_{u}\left(1+\sin\left(\frac{1}{2}\nu t^{2}\right)\right)$$ (11)

where *u*_0_ is the motor torque corresponding to the threshold force value, *A*_*u*_ is desired torque amplitude, and *ν* is the oscillation frequency variation factor. This coefficient was calculated as $\nu =\frac{w_{f}-w_{0}}{t_{f}}$ where *w*_0_ is the initial radial frequency, *w*_*f*_ is the final frequency and *t*_*f*_ is experiment duration. The experiment lasted for 120 s with the linear frequency changing from 1 to 10 Hz, while the initial torque was *u*_0_ = 19 mNm and desired torque amplitude was *A*_*u*_ = 47 mNm. Since the proposed TSA-based joint is a nonlinear device, we conducted these experiments at 9 different values of the angle *φ* between 15° and 152°. We collected the same sensor data with the previous experiment.

### 4.2 Results

**FIGURE 6:**
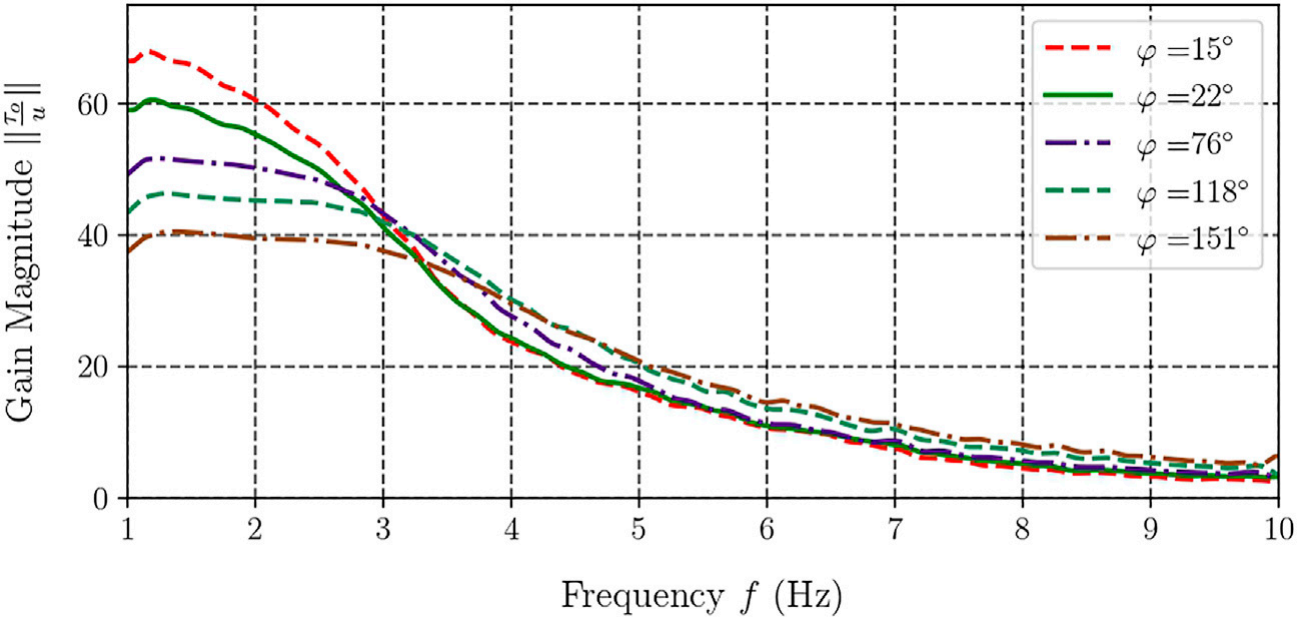
Frequency response of the gain from motor torque to handle torque.

The results for five selected handle angles are shown in Figure 6. One can note from the resulting graph that the curves demonstrate similar behaviour, their values dropping monotonically with the increase in motion frequency. The initial values of *K* calculated with Eq. 10 drop with increasing twisting, much like the curve plotted in Figure 5 for the pseudo-static case.

**FIGURE 7:**
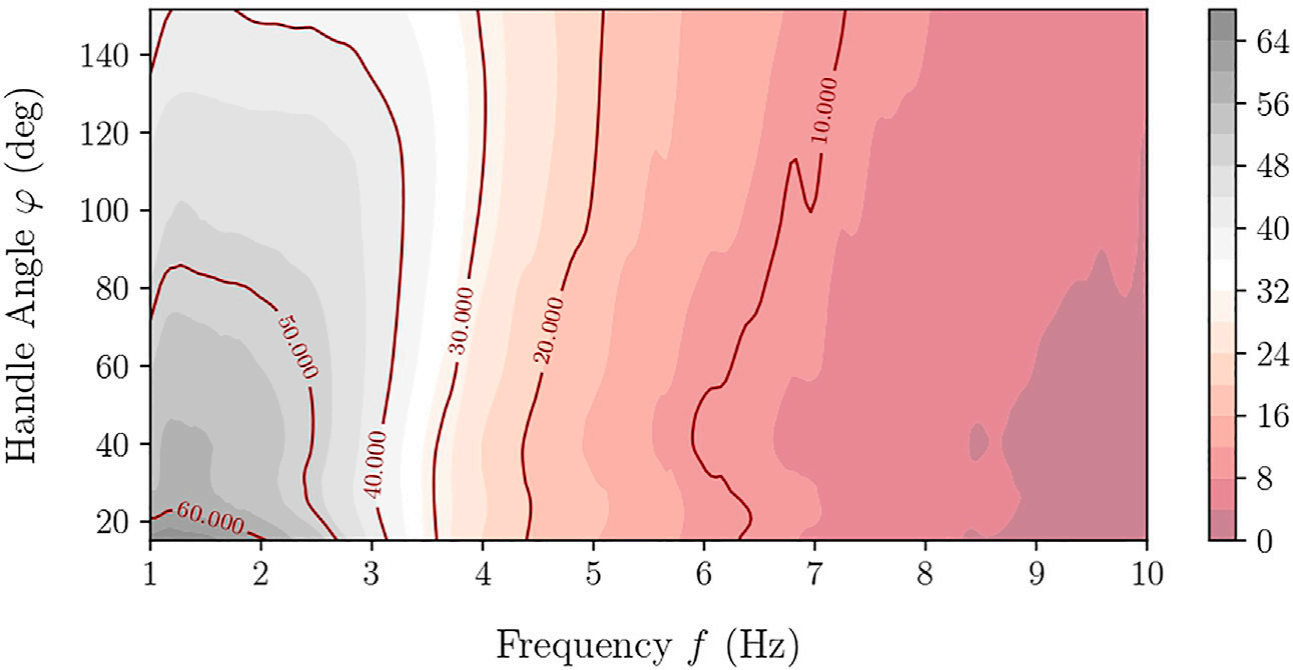
Contour plot of the frequency response of the setup for different angles of the handle.

**FIGURE 8:**
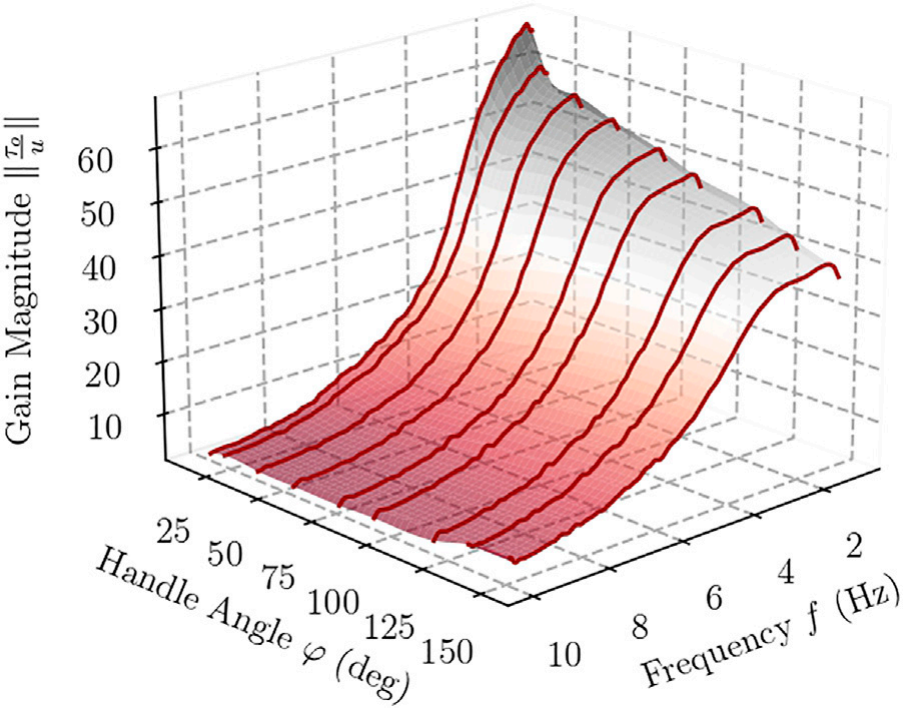
Interpolating surface along the frequency response experiments for different handle angles.

Figures 7, 8 feature a contour plot of the transmission ratio and the corresponding three-dimensional graph in which *K* is plotted with respect to the handle’s angle and frequency. In these plots, one can observe a steady decrease of the gain *K* with frequency, from about 60 for small angles of the handle to below 10 at high frequencies, irrespective of angle *φ*. Once the frequency exceeds 4 Hz, the device exhibits nearly identical gain magnitude *K* regardless of the handle’s angle.

## 5 Testing haptic interaction

To test the haptic feedback capabilities of our device, we implemented a virtual wall. Multiple tests were conducted with the virtual wall’s stiffness coefficient in the range of 1–2 Nm/rad. In the following, we describe the virtual wall rendering technique we propose for our TSA-based interface and the corresponding testing results.

### 5.1 Implementing virtual wall with the TSA-based interface

The handle torque generated by the virtual wall, *τ*_*w*_, was set as follows:

$$\tau_{w}=K_{w}\left(\varphi_{0}-\varphi \right),\;if\;\varphi >\varphi_{0}\;\text{OR}\;\tau_{w}=0\;otherwise,$$ (12)

with the stiffness coefficient and the position of the virtual wall defined as *K*_*w*_ and *φ*_0_, respectively. Usage of the TSA for the handle motion does not allow direct application of the haptic wall’s torque, *τ*_*w*_. Therefore, to implement such a wall with our TSA-based interface it is necessary to continuously adjust the handles angular position based on the (estimated or measured) torque applied at the handle by a user interacting with the haptic wall:

$$\varphi =\varphi_{d}=\varphi_{0}-\frac{\hat{\tau }}{K_{w}}$$ (13)

where *φ*_*d*_ is desired handle’s angular orientation, such that the haptic wall Eq. 12 is implemented based on the estimated/measured user-handle interaction torque $\hat{\tau }$ . For our device, an estimate of the torque applied to the handle by the user was obtained using the handle’s load cell measurements fed through a low-pass filter to reduce sensor noise.

Once the force regulated set point *φ*_*d*_ is found one can use it as a reference point in any servo-like controller. In this study, for simplicity we have selected the control algorithm similar to the TSA Jacobian-based technique developed and tested by Nedelchev et al. (2019):

$$\left\{\begin{array}{c} u_{s}={\hat{J}}_{d}^{-1}\left({\dot{\varphi }}_{d}+k_{p}\left(\varphi_{d}-\varphi \right)\right)\;\\ u=k_{s}\left(u_{s}-\dot{\theta }\right)+k_{i}\int_{t_{0}}^{t}\left(u_{s}-\dot{\theta }\right)d\tau \; \end{array}\right.$$ (14)

where *u*_*s*_ is the Jacobian-based speed reference for the TSA actuator, *k*_*p*_ is a positive gain that defines the speed of response, ${\hat{J}}_{d}$ is the estimate of the device’s Jacobian. The speed reference, *u*_*s*_, is then fed to a conventional PI controller with the gains *k*_*s*_, *k*_*i*_ to produce the torque *u* which is tracked by the motor. Implementation of the laws Eq. 13 and Eq. 14 allows tracking required virtual wall torque Eq. 12 at the device’s handle.

### 5.2 Experiment and results

We conducted several experiments in which the user interacted with the virtual wall implemented with the aforementioned control law Eq. 14. During the interaction, the user’s task was to repetitively press and release the device’s handle which resulted in the increase and decrease of the torque, simulating interaction with a virtual wall.

**FIGURE 9:**
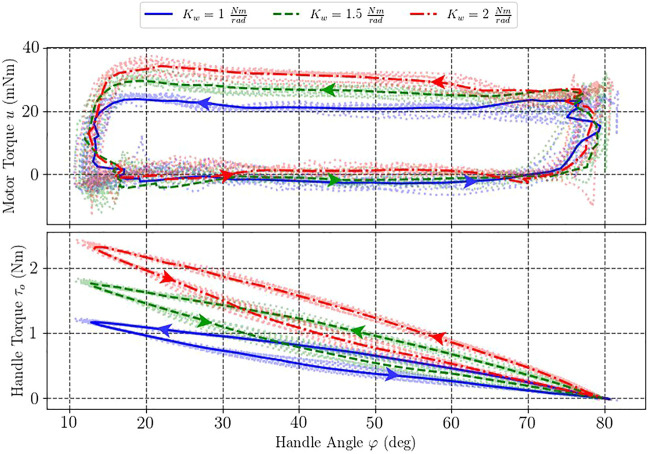
Results for haptic virtual wall rendering. Comparison of handle and motor torque values at different stiffness coefficients of the virtual wall.

The experimental results are shown in Figure 9. One can note that the actual measured torque on the handle increased almost linearly, as expected according to Eq. 13, with some hysteresis which is likely to appear due to control and filtering transients. The maximum handle torque for the stiffness coefficient of *K*_*w*_ = 1 Nm/rad was *τ*_max_ = 1.2 Nm, while for 1.5 Nm/rad and 2 Nm/rad the respective torque values were measured at 1.8 Nm and 2.4 Nm, correspondingly. We performed 10 repetitive arm movements during the experiment. The standard deviation of the handle force measurements was 0.03, 0.05, and 0.06 Nm for *K*_*w*_ = 1.0, 1.5, and 2.0 Nm/rad, respectively for each experiments.

In contrast, one can notice from Figure 9 that the developed motor torque did not increase two-fold like the handle torque and, in fact, remained nearly constant for each particular experiment. Specifically, average motor torque values during string contraction (top parts of the curves) were equal to 22, 25, and 28 mNm for the virtual wall with stiffness coefficients of *K*_*w*_ = 1.0, 1.5, and 2.0 Nm/rad, respectively. This was achieved thanks to a nonlinear relationship between the motor and handle angles (TSA transmission ratio). In particular, when the handle was pressed at higher values of joint angles (60°–80°) which corresponded to the region of significant string contraction (lower transmission ratios), the handle would then be displaced into the region requiring smaller string contraction magnitudes (higher transmission ratio). This region requires the motor to exert *smaller* torques to support the same force, however, since the desired handle torque increased in a spring-like fashion, these two effects worked against each other.

One can also notice significant hysteresis in the motor torque readings due to the unidirectional nature of the pulling tendons: in the pressing mode (bottom side of the curves), the returning springs were helping the user while during the release the TSA was subject to both handle and spring forces. Torque hysteresis also increased with the stiffness coefficient.

## 6 Conclusion

This paper reports on the design and experimental evaluation of a TSA-based human-machine wrist interface. The device employs twisted strings to drive a pulley with a handle attached and through this can transmit high torques to the user’s wrist.

We have performed experimental characterization of the device’s kinematics and statics while also evaluating its dynamical force transmission properties in a series of experiments with frequencies reaching 10 Hz. Experiments aimed at the evaluation of device kinematics reported an RMS error of approximately 2.15%. To the best of our knowledge, this is the first evaluation of its kind of a TSA-based wrist haptic interface, studying its force transmission capabilities at high frequencies.

The device produced peak handle torques of over 7 Nm (near the initial configuration) while being capable of generating short-term torques in excess of 1 Nm for the whole workspace and frequencies under 8 Hz. In addition, the current version of the device can generate torques of over 400 mNm for the whole range of angles and frequencies without motor overheating, the value which can be easily increased by using a more powerful motor or strings of smaller radius.

We have performed a preliminary user evaluation of the developed device with a virtual haptic wall interaction test for different stiffness levels. As the nonlinear transmission ratio of the TSA increased in the lower contraction region, the motor needed to generate smaller torques to support high handle torques, which is beneficial for the design and cost aspects as smaller and simpler electric motors can be used.

In the future, we plan to conduct a more thorough evaluation of the device in a variety of user interaction scenarios. Additionally, making a sturdier version of the prototype (by replacing some components made of plastic with metal parts) will increase the stiffness of the mechanism and will improve the accuracy of the system’s model. Furthermore, the load cells and the linear encoder used in the prototype can be removed in the future designs as they were used for the devices’ evaluation purpose only. The other potential research direction is the development of a bidirectional device that employs an additional TSA mechanism instead of passive springs. That would contribute to improved control over the device’s handle position and speed and would support a much larger range of achievable stiffness coefficients of the handle.

## Data Availability

The raw data supporting the conclusion of this article will be made available by the authors, without undue reservation.
